# CD20, CD3, placental malaria infections and low birth weight in an area of unstable malaria transmission in Central Sudan

**DOI:** 10.1186/1746-1596-8-189

**Published:** 2013-11-18

**Authors:** Samah E Batran, Magdi M Salih, Elhassan M Elhassan, Ahmed A Mohmmed, Ishag Adam

**Affiliations:** 1 Faculty of Medical Laboratory Sciences, University of Khartoum, Khartoum, Sudan; 2 Faculty of Medicine, University of Gezira, Medani, Sudan; 3 Faculty of Medicine, Ribat University, Khartoum, Sudan; 4 Faculty of Medicine, University of Khartoum, Khartoum, Sudan

**Keywords:** Malaria, Pregnancy, Low birth weight, CD20, CD3, Sudan

## Abstract

**Background:**

Malaria during pregnancy is the main cause of low birth weight (LBW) in the tropics. There are few studies concerning B and T lymphocyte infiltrates in placental malaria infections or their potential association with LBW babies.

**Methods:**

A case–control study was conducted at the Medani Hospital, Central Sudan. Cases were women who had LBW deliveries (infants weighed < 2,500 g) and controls were parturient women with normal birth weight babies. Sociodemographic and medical characteristics were gathered from both groups of women using questionnaires. Cases and controls were investigated for malaria using microscopic blood film analysis, placental histology, and immunohistochemistry for detection of B (CD20) and T lymphocytes (CD3).

**Results:**

The two groups (97 in each arm) were well matched in their basic characteristics. There were no malaria-positive blood films in either the cases or the controls. Twenty-nine (30.0%) *vs.* 24 (24.7%), P = 0.519 of the cases *vs.* the controls had placental malaria infections on histological examination. Three (3.1%), two (2.1%) and 24 (24.7%) *vs.* two (2.1%), two (2.1%) and 20 (20.6%) of the placentae showed evidence of acute, chronic and past malarial infections on histopathological examination of the two groups (case–control), respectively, while 68 (70.1%) *vs.* 73 (75.3%) of them showed no signs of infection; P = 0.420. Women with placental malaria infections had significantly fewer CD20 cell infiltrates [6 (11.3% vs. 95 (67.4%), P < 0.001)] and higher numbers of CD3 cell infiltrates [50 (94.3%) vs. 42 (29.8%), P < 0.001] than those without placental malaria infection. Logistic regression analysis showed that neither placental malaria infections nor CD3 or CD20 were associated with LBW.

**Conclusions:**

Significantly higher rates of CD3 T cells and lower rates of CD20 B cells were found in women with placental malaria infections compared with those without such infections. Neither placental malaria infection nor CD3 or CD20 are associated with LBW.

**Virtual slides:**

http://www.diagnosticpathology.diagnomx.eu/vs/6879723961063755

## Background

Malaria is a big public health problem in tropical countries, especially in sub-Saharan African countries where around 125 million pregnant women live in malaria-endemic areas and 32 million of these are at risk of the disease [1,2]. Malaria during pregnancy can lead to maternal and perinatal adverse effects such as anemia [3] and low birth weight (LBW) delivery [4], the latter of which is the main risk for neonatal and infant morbidity and mortality [5,6].

During pregnancy, adhesion of *Plasmodium falciparum*-infected erythrocytes to the syncytiotrophoblast leads to parasite sequestration in the intervillous space. The parasite adheres specifically to chondroitin sulfate-A expressed on the syncytiotrophoblast [7]. The increased susceptibility of pregnant women to malaria is thought to be a result of pregnancy-related immunomodulation, which is more active in the placenta than in the peripheral blood [8]. Inflammatory responses in the placenta may lead to functional damage in the placental villi, thus disturbing feto-maternal exchange and leading to LBW [5]. Histological studies on placental malaria have shown that *P. falciparum*-infected placentae are characterized by an increase in inflammatory cells and that such cells are mainly monocytes and macrophages with a smaller population of granulocytes and lymphocytes [9]. Few studies have investigated immunological responses related to placental cell infiltrates [10].

Malaria during pregnancy is a long-standing health problem in Sudan where pregnant women are susceptible to malaria during pregnancy regardless of their age or parity [3]. In Sudan, malaria is associated with LBW [4], which is a leading cause of high perinatal mortality [11]. The current study was conducted to investigate B and T lymphocytes (using CD 20 and CD3 as markers) and their association- if any- with malarial infection and LBW. Our findings add to the current scientific knowledge about malaria [12], pregnancy-related malaria, LBW and placental cell infiltrates [4,13].

## Methods

A case–control study was conducted during the rainy and post rainy seasons during August to December 2011 at the labor ward of the Medani Maternity Hospital, Central Sudan. Central Sudan is characterized by unstable malaria transmission and *P. falciparum* is the sole malaria parasite species in the area: malaria transmission occurs during the rainy (July –September) and post-rainy seasons [14]. Medani Maternity Hospital is a tertiary hospital for women who receive antenatal care at the hospital, or are referred from other clinics and hospitals, and for women who live close to the hospital facility. Women with high-risk pregnancies are referred to the hospital. However, the referral criteria are not strictly adhered to and many women without a high-risk pregnancy deliver at the hospital.

A sample size calculation was made to provide over 80% power to detect a difference of 5% at α = 0.05. This was based on the assumption that 10% of women might not respond or have incomplete data. In this study, a case represents a woman who had a LBW delivery (<2,500 g). A consecutive woman who delivered next to the case and had a normal birth weight baby at delivery (≥ 2,500 g) was taken as a control for each case. Women pregnant (case or controls) with twins and those with hypertension, diabetes mellitus or antepartum hemorrhage were excluded from the study. After obtaining signed informed consent, women in the case and control groups were enlisted to participate in the study. Information on sociodemographics, obstetrics history, medical characteristics and antenatal attendance was gathered through structured pretested questionnaires. Women in both groups were asked if they used bed nets and if they had experienced malaria infections in the index pregnancy. Body mass index was calculated by measuring maternal weight and height, which was expressed as weight (kg)/height (m)^2^. Babies were weighed immediately following birth to the nearest 10 g on a Salter scale. Scales were checked for accuracy on a weekly basis. The gender of each baby was recorded.

### Giemsa-stained blood smears and light microscopy

Maternal, placental and cord blood films were prepared and the resultant slides were stained with 10% Giemsa. The numbers of asexual parasites were counted per 200 leukocytes assuming a leukocyte count of 8,000 leukocytes/μl (for thick films) or per 1,000 red blood cells (for thin films). Blood films were considered negative if no parasites were detected in 100 oil immersion fields of a thick blood film. Films were counted and double-checked blindly by an expert microscopist. Maternal hemoglobin concentrations were estimated using a HemoCue hemoglobinometer (HemoCue AB, Angelhom, Sweden).

### Placental histology

Placental histology was conducted as described in previous work [4,12,13]. In brief, a sample of approximately three cm^3^ was removed from the maternal surface in an off-center position, at a distance of half way between the umbilical cord and the edge of the placenta. Once collected, each biopsy sample was placed in 25 mL of 10% neutral buffered formalin. Buffer was used to prevent formalin pigment formation, which has similar optical characteristics and polarized light activity as malaria pigment [15]. Placental biopsy samples were stored at room temperature in Medani until transportation to Khartoum, where the histology was performed. The biopsy samples were processed by embedding them in paraffin wax using standard techniques. In every case, the 4 mm thick paraffin sections were stained with hematoxylin-eosin and Giemsa stains. Placental malaria infections were characterized as previously described by Bulmer, et al. 1993 [16]: uninfected (no parasites or pigment), acute (parasites in intervillous spaces), chronic (parasites in maternal erythrocytes and pigment in fibrin, or cells within fibrin and/or chorionic villous syncytiotrophoblast or stroma), and past (no parasites and pigment confined to fibrin or cells within fibrin). Slides were read by a pathologist who remained blind to the clinical characteristics of the participants and the arms of the study.

### Immunohistochemical methods

**Figure 1:**
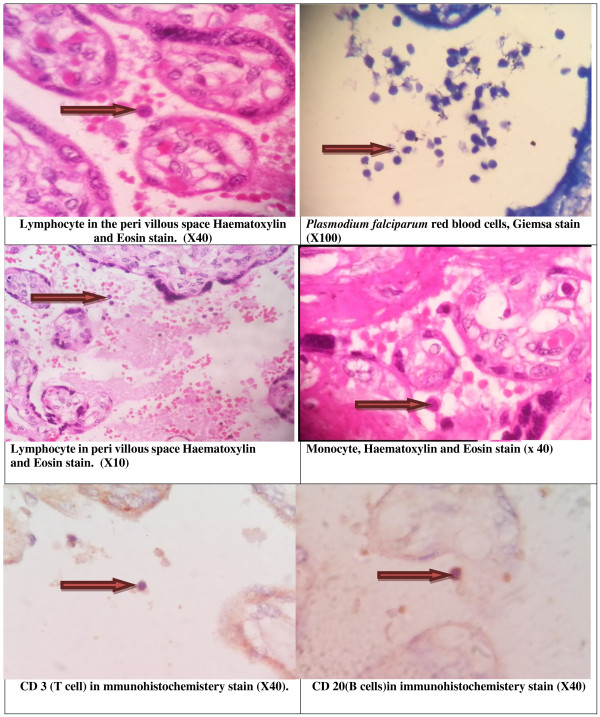
Placental malaria and lymphocytes infiltrations.

Details of the immunohistochemical methods used can be found elsewhere [4,12,13]. Briefly, immunohistochemical analysis was performed in neutral formalin-fixed, paraffin-embedded tissue using EnVision™ FLEX Solution according to the manufacturer’s instructions (http://www.ihcworld.com/products/IHC-Tek-Reagent.htm). To summarize, 3-mm sections were deparaffinized and hydrated through xylene and graded alcohols, and peroxidase was blocked for 5 min in 0.03% H_2_O_2_ containing sodium azide. The slides were then incubated with a primary antibody against CD 20 or CD3 for 40 min. A peroxidase-labeled polymer was applied to the slides for 40 min. After washing in TBS, the slides were incubated with a solution containing diaminobenzidine substrate chromogen, washed in distilled water, counterstained with hematoxylin, re-washed, dehydrated, and mounted. A water bath was used for heat-induced epitope retrieval with the antibodies [17]. B and T lymphocyte inflammatory cell quantification was performed with an Olympus microscope at a magnification of × 40 using an eyepiece lens (Figure 1). The numbers of B and T CD20 and CD3 cells were expressed as the log of the mean values.

### Statistics

Data were analyzed using SPSS for windows version 16.0. Student’s *t*-test and *X*^2^ were used to compare means and proportions between the groups, respectively. Univariate and multivariate analyses were performed where malaria infection was the dependent variable and expected risk factors (e.g., age, parity, and education, amongst others) were the independent variables. Another model was set for anemia and LBW. Odds ratios (OR) and 95% confidence intervals (CI) were calculated and statistical significance was defined as *P* < 0.05.

## Results

There were no significant differences between the two groups (case or control) in their age, parity, level of education, residence, antenatal care attendance, bed net usage and other sociodemographic characteristics, Table 1.

**Table 1:** Sociodemographic characteristics of the cases and controls

**Variable**	**Cases (n = 97)**	**Controls (n = 97)**	**P**
*Mean (SD) of*			
Age	25.3(5.4)	26.8(6.1)	0.07
Parity	1.4(1.4)	1.8(1.7)	0.09
Body mass index	24.4(2.6)	23.8(3.2)	0.175
Hemoglobin, g/dl	10.0(1.0)	9.8(1.0)	0.152
*Number (percentage) of*			
Rural residency	67(69.1)	65(67.0)	0.878
Maternal education > secondary level	76(90.5)	86(78.4)	0.056
Lack of antenatal care	12(0.123)	14(14.4)	0.381
Using bed nets	14(14.4)	14(14.4)	1.000
Male gender	51(0.525)	51(0.525)	1.000

### Malaria infections

No *P. falciparum*-positive blood films were obtained for maternal peripheral blood, placenta or cord samples in either the cases or the controls. Twenty-nine (30.0%) *vs.* 24 (24.7%) (P = 0.519) of the cases *vs.* controls had placental malaria infections on histological examination. Three (3.1%), two (2.1%) and 24 (24.7%) *vs.* two (2.1%), two (2.1%) and 20 (20.6%) of the placentae showed evidence of acute, chronic or past malarial infections on histopathology examination of the two groups (case–control), respectively, while 68 (70.1%) *vs.* 73 (75.3%) of them showed no signs of infection; P = 0.420, Table 2. In the logistic regression, age and parity were not associated with placental malaria infection. Anemic women were less likely to have malaria infections, Table 3.

**Table 2:** Placental infections, CD 20 and CD 3 in cases and controls

**Variable**	**Cases (n = 97)**	**Controls (n = 97)**	**P**
Total malaria infection	29(30.0)	24(24.7)	0.519
Acute	3(3.1)	2(2.1)	0.650
Chronic	2(2.1)	2(2.1)	0.999
Past	24(24.7)	20(20.6)	0.492
Negative	68(70.1)	73(75.3)	0.420
CD20	46(47.4)	55(56.7)	0.250
CD3	47(48.5)	45(46.5)	0.886
Either CD20 or CD3	75(77.3)	76(78.4)	1.000
Both CD20 and CD3	18(18.6)	24(24.7)	0.384

**Table 3:** Factors associated with malaria infections as determined by univariate and multivariate analyses

	**Univariate analysis**			**Multivariate analysis**		
**Variable**	**OR**	**95% CI**	**P**	**OR**	**95% CI**	**P**
Age	0.9	0.9–1.0	0.152	0.9	0.8–1.0	0.781
Parity	0.8	0.7–1.0	0.185	0.9	0.6–1.3	0.954
Residence	0.6	0.3–1.4	0.696	0.6	0.2–1.4	0.260
Maternal education < secondary level	0.7	0.2–1.8	0.485	0.4	0.1–1.4	0.161
Lack of antenatal care	1.3	0.6–2.5	0.242	1.1	0.4–3.1	0.801
Body mass index	1.0	0.9–1.0	0.668	1.0	0.9–1.1	0.524
Not using bed nets	1.4	0.6–3.5	0.350	0.9	0.3–2.9	0.982
Anemia	0.3	0.1–0.8	0.016	0.2	0.08–0.75	0.013

### CD20 and CD3 in malaria infections

In comparison to women without placental malaria infections, women with such infections had significantly fewer CD20 cell infiltrates [6 (11.3%) *vs*. 95(67.4%), P < 0.001)] and significantly higher numbers of CD3 cell infiltrates [50 (94.3%) *vs*. 42 (29.8%), P < 0.001].

Acute, chronic and past malaria infections had 1, 0, 5 CD20 and 5, 4, 41 CD3 cell infiltrates, respectively.

In the logistic regression, age and parity were not associated with CD20 infiltration; however, malaria infections were less likely to have cell infiltrates, and CD3 was significantly associated with CD20, Table 4. Likewise, while age and parity were not associated with CD3 infiltration, placental malaria infection and CD20 were significantly associated with CD3, Table 4.

**Table 4:** Factors associated with CD20 and CD3 at Medani hospital as determined by logistic regression analysis

	**CD 20**			**CD 3**		
**Variable**	**OR**	**95%**	**P**	**OR**	**95%**	**P**
Age	1.0	0.9–1.1	0.201	1.01	0.9–1.1	0.702
Parity	0.8	0.6–1.1	0.368	0.92	0.7–1.2	0.945
Residence	1.4	0.6–3.2	0.380	0.9	0.4–2.0	0.986
Maternal education < secondary level	0.7	0.2–2.4	0.680	0.7	0.2–2.0	0.605
Lack of antenatal care	1.0	0.3–2.6	0.980	1.2	0.5–2.6	0.652
Body mass index	0.9	0.8–1.1	0.832	1.0	0.9–1.1	0.555
Anemia	0.02	0.009–0.098	<0.001	3.6	0.9–14.4	0.067
Malaria infection	0.6	0.1–2.1	0.482	98.5	23.1–418.3	<0.001
CD20	-			3.7	1.4–9.8	0.007
CD3	3.6	1.3–9.6	0.009	-		

In women where CD20 and CD3 were detected, the log of the mean (SD) of CD20 was not statistically different [2.5 (3.7) *vs*. 2.6 (2.2), P = 0.928 cells]. However, the log of the mean (SD) for CD3 was significantly higher in women with placental infections (n = 50) than in women who had no placental infections (n = 42), [5.1 (3.0) *vs.* 1.6 (2.4) cells, P < 0.001].

### CD20 and CD3 in cases and controls

CD20 and CD3 were detected in 46 (47.4%) *vs.* 55 (56.7%), P = 0.250 and 47 (48.5%) *vs.* 45 (46.5%), P =0.886 in cases and controls, respectively. Both CD20 and CD3 were detected in 18 (18.6%) *vs*. 24 (24.7%), P = 0.384 of the cases and controls, respectively. Likewise, 75 (77.3%) and 76 (78.4%), P =1.000 of the cases and controls had either CD20 or CD3 infiltrates, Table 2.

### Effects of malaria infection, CD20, and CD3 on birth weight and anemia

The mean (SD) for birth weight was 2,375.8 (1548.0) *vs.* 3,314.6 (3492) g, P < 0.001 in the cases and controls, respectively. There was no significant difference in the mean (SD) of the birth weight between those who had placental malaria infections (as determined by histology) (in both groups, n = 54) and those who did not (n = 140) [2845.3 (576) *vs.* 2841.8 (530) g, P =0.968]. Likewise, the mean (SD) of the birth weights was no different in women who had CD20 cell infiltrates (in both groups, n = 101) than in those who had no evidence of CD20 cell infiltrates [2879.0 (536) *vs.* 2803.8 (548) g, P = 0.337] or those who had CD3 (in both groups = 92) [2854.4 (560) *vs.* 2832.4 (528) g, P = 0.779].

In this multivariate analysis, neither placental *P. falciparum* infection nor CD3 or CD20 were associated with LBW or with anemia, Table 5.

**Table 5:** Factors associated with anemia and low birth weight as determined by logistic regression analyses

	**Anemia**			**Low birth weight**		
**Variable**	**OR**	**95%**	**P**	**OR**	**95%**	**P**
Age	1.0	0.8–1.1	0.863	0.9	0.8-1.0	0.961
Parity	1.0	0.6–1.1	0.800	0.9	0.6-1.2	0.979
Residence	0.4	0.1–1.4	0.479	0.5	0.2-1.2	0.183
Maternal education < secondary level	0.4	0.1–1.6	0.221	2.1	0.7-6.4	0.154
lack of antenatal care	4.8	1.3–17.4	0.017	1.3	0.5-3.2	0.528
Body mass index	0.8	0.7–1.0	0.857	1.0	0.9-1.2	0.172
Not using bed nets	1.9	0.6–6.5	0.262	0.6	0.2-1.7	0.372
Anemia	-			0.7	0.2-2.0	0.536
Gender	-			1.0	0.5-2.0	0.756
Malaria	-			1.0	0.3-2.9	0.891
CD3	0.9	0.3–2.5	0.916	1.0	0.8-1.3	0.439
CD20	1.6	0.5–4.5	0.336	1.1	0.9-1.4	0.226

## Discussion

The main findings of the current study were as follows: Placental malaria infections (the majority of which were past infections) were detected in 27.0% of the women, regardless of their age or parity. Placental malaria infections had fewer and higher numbers of CD20 and CD3, respectively. Neither placental infections nor CD20 and CD3 cell infiltration had an effect on LBW or anemia. In the same hospital (Medani Maternity Hospital), placental malaria infections were detected in placentae regardless of the age or parity of the women and placental malaria infections (detected by histology) were found not to be associated with LBW [4]. We have recently observed that monocytes and macrophages (CD68) were detected in 29 (31.2%) of 93 placentae in Eastern Sudan regardless of the age or parity of the women [4]. Furthermore, as was also observed in the current study, cell infiltrates were not associated with malaria infections: there were significantly higher rates of monocytes and macrophages detected in placentae with malaria infections (47.8% *vs*. 25.7%) and monocytes and macrophage infiltrates were not associated with birth weight [4]. In an area of intense malaria transmission in Tanzania, placental malaria infections (mainly in primigravidae) showed the most significant increases in all inflammatory cell types with the exception of NK cells, with monocytes and macrophages representing the major cell population in the infiltrates [10]. Yet, it has been observed that inflammatory responses are particularly marked in chronic placental malaria infections, unlike past placental infections, and that infiltrates in chronic infections are associated with reduced birth weight [10]. Likewise, Ismail and colleagues [9] observed that primigravidae had higher placental parasitaemias as well as higher frequencies of chronic infections and inflammatory cell infiltrates than multiparae. They also observed that chronic malaria infections exhibited significant levels of inflammatory cell infiltrates and acute infections showed small increases in inflammatory cell infiltrates, while those with past infections exhibited no increases in cell infiltrates. Therefore, it is likely that the discrepancies in placental malaria infections and cell infiltrates between primigravidae and multigravidae identified in the above studies, but not in the current study, related to differences in the intensity of malaria transmission between different settings. This is because it is expected that pregnant women will have low immunity in areas with unstable malaria transmission.

Interestingly, in the current study, in comparison to women without placental malaria infections, women with such infections had significantly fewer B cell (CD20) infiltrates, and larger numbers of T cell (CD3) infiltrates. This finding supports a published study where Ordi, et al. [10] observed a marked increase in the number of monocytes, macrophages and cytotoxic T cells in the intervillous spaces of placentas infected with malaria parasites compared with non-infected placentas.

Consequently, our findings on B cells and T cells, as described in the current study may explain why pregnant women have increased susceptibility to malaria, which is thought to result from pregnancy-related immunomodulation and a Th1/Th2 shift to decreased Th1-type cytokines and increased Th2-type cytokines to prevent rejection of the fetal allograft [18,19]. There is no obvious explanation of the absence of association between B and T cells and LBW in this study, yet unforeseen confounder might be there. The Th1/Th2 shift towards increased Th2-type cytokines during pregnancy and susceptibility to malaria infections was observed in previous studies in Eastern and Central Sudan and was attributed to the hormonal changes [20,21]. The pregnancy-related immune-modulation appears more likely to be placental in origin as opposed to that taking place in the peripheral blood [8]. Inflammatory responses in the placenta can lead to functional damage to the placental villi and disturbed normal feto-maternal exchange, thereby leading to low birth weight [5].

## Conclusions

Significantly higher rates of CD3 T cells and lower CD20 B cells were detected in malaria-infected placentae than in placentae without such infections. Neither placental malaria infections nor cellular infiltrates were associated with parity or led to reductions in birth weight.

## Competing interests

The authors’ declare that they have no competing interests.

## Authors’ contributions

SB and IA designed the study and EME conducted the clinical work. MMS and AAM conducted the laboratory work. IA and SB participated in the statistical analyses. All the authors’ have approved the final paper.

## References

[B1] WHO World malaria report 2010 2010 Geneva, Switzerland: World Health Organization 2010

[B2] Dellicour S Tatem AJ Guerra CA Snow RW ter Kuile FO Quantifying the number of pregnancies at risk of malaria in 2007: a demographic study PLoS Med 2010 8 1000 1221 10.1371/journal.pmed.1000221 PMC2811150 20126256

[B3] Adam I Elhassan EM Haggaz AE Ali AA Adam GK A perspective of the epidemiology of malaria and anaemia and their impact on maternal and perinatal outcomes in Sudan J Infect Dev Ctries 2011 8 83 87 21389586 10.3855/jidc.1282

[B4] Mohammed AH Salih MM Elhassan EM Mohmmed AA Elzaki SE El-Sayed BB Adam I Submicroscopic plasmodium falciparum malaria and low birth weight in an area of unstable malaria transmission in Central Sudan Malar J 2013 8 172 178 10.1186/1475-2875-12-172 23714259 PMC3671167

[B5] Menendez C Ordi J Ismail MR Ventura PJ Aponte JJ Kahigwa E Font F Alonso PL The impact of placental malaria on gestational age and birth weight J Infect Dis 2000 8 1740 1745 10.1086/315449 10823776

[B6] Steketee RW Nahlen BL Parise ME Menendez C The burden of malaria in pregnancy in malaria-endemic areas Am J Trop Med Hyg 2001 8 28 35 11425175 10.4269/ajtmh.2001.64.28

[B7] Reeder JC Hodder AN Beeson JG Brown GV Identification of glycosaminoglycan binding domains in *Plasmodium falciparum* erythrocyte membrane protein 1 of a chondroitin sulfate A-adherent parasite Infect Immun 2000 8 3923 3926 10.1128/IAI.68.7.3923-3926.2000 10858204 PMC101668

[B8] Diouf I Fievet N Doucoure S Ngom M Gaye A Dumont A Ndao CT Le Hesran JY Chaouat G Deloron P Monocyte activation and T cell inhibition in plasmodium falciparum-infected placenta J Infect Dis 2004 8 2235 2242 10.1086/420791 15181571

[B9] Ismail MR Ordi J Menendez C Ventura PJ Aponte JJ Kahigwa E Hirt R Cardesa A Alonso PL Placental pathology in malaria: a histological, immunohistochemical and quantitative study Hum Pathol 2000 8 85 93 10.1016/S0046-8177(00)80203-8 10665918

[B10] Ordi J Menendez C Ismail MR Ventura PJ Palacín A Kahigwa E Ferrer B Cardesa A Alonso PL Placental malaria is associated with cell-mediated inflammatory responses with selective absence of natural killer cells J Infect Dis 2001 8 1100 1107 10.1086/319295 11237836

[B11] Hassan AA Abubaker MS Radi EA Adam I Education, prenatal care, and poor perinatal outcome in Khartoum, Sudan Int J Gynaecol Obstet 2009 8 1 66 67 10.1016/j.ijgo.2008.10.026 19062014

[B12] Kashif AH Adam GK Mohmmed AA Elzaki SE AbdelHalim AM Adam I Reliability of rapid diagnostic test for diagnosing peripheral and placental malaria in an area of unstable malaria transmission in Eastern Sudan Diagn Pathol 2013 8 59 10.1186/1746-1596-8-59 23587371 PMC3640898

[B13] Salih MM Mohammed AH Mohmmed AA Adam GK Elbashir MI Adam I Monocytes and macrophages and placental malaria infections in an area of unstable malaria transmission in eastern Sudan Diagn Pathol 2011 8 80 83 10.1186/1746-1596-6-80 21929772 PMC3182959

[B14] Malik EM Atta HY Weis M Lang A Puta C Lettenmaier C Sudan Roll Back Malaria Consultative Mission: Essential Actions to Support the Attainment of the Abuja Targets. Sudan RBM Country Consultative Mission Final Report 2004 Geneva: Roll Back Malaria Partnership

[B15] Bulmer JN Rasheed FN Morrison L Francis N Greenwood BM Placental malaria. II. A semi-quantitative investigation of the pathological features Histopathology 1993 8 3 219 225 10.1111/j.1365-2559.1993.tb00111.x 8495955

[B16] Bulmer JN Rasheed FN Francis N Morrison L Greenwood BM Placental malaria. I. Pathological classification Histopathology 1993 8 211 218 10.1111/j.1365-2559.1993.tb00110.x 8495954

[B17] Battifora H Alsabeh R Jenkins KA Gown A Weinstein RS, Graham AR, Andreson RE Epitope retrieval unmasking in immunohistochemistry Advances in pathology and laboratory medicine 1995 St. Louis: CV Mosby 101 18

[B18] Krishnan L Guilbert LJ Wegmann TG Belosevic M Mosmann TR T helper 1 response against Leishmania major in pregnant C57BL/6 mice increases implantation failure and fetal resorptions. Correlation with increased IFN-g and TNF and reduced IL-10 production by placental cells J Immunol 1996 8 653 662 8543817

[B19] Wegmann TG Lin H Guilbert L Mosmann TR Bidirectional cytokine interactions in the maternal–fetal relationship: is successful pregnancy a Th2 phenomenon? Immunol Today 1993 8 353 356 10.1016/0167-5699(93)90235-D 8363725

[B20] Bayoumi NK Bakhet KH Mohmmed AA Eltom AM Elbashir MI Mavoungou E Adam I Cytokine profiles in peripheral, placental and cord blood in an area of unstable malaria transmission in eastern Sudan J Trop Pediatr 2009 8 233 237 10.1093/tropej/fmn062 18614592

[B21] Bayoumi NK Elhassan EM Elbashir MI Adam I Cortisol, prolactin, cytokines and the susceptibility of pregnant Sudanese women to Plasmodium falciparum malaria Ann Trop Med Parasitol 2009 8 111 117 10.1179/136485909X385045 19208295

